# Establishment and characterization of a novel ‘double‐hit’ follicular lymphoma cell line, FL‐SJC

**DOI:** 10.1111/jcmm.15425

**Published:** 2020-05-27

**Authors:** Min Chen, Guoxiong Jiang, Yichen Liu, Dongya Li, Tiantian Li, Jie Peng, Qian Jiang, Haiyan You, Rong Ba, Jinlan Pan, Mei Li, Weiguo Long, Jinsong Yan, Yan Zhu, Yun Wang, Xiaodong Xi, Jianhua Mao, Xiaofeng Shi

**Affiliations:** ^1^ Affiliated Hospital of Jiangsu University Zhenjiang China; ^2^ Jiangdu People’s Hospital Yangzhou China; ^3^ Affiliated Hospital of Suzhou University Suzhou China; ^4^ State Key Laboratory of Medical Genomics Shanghai Institute of Hematology Ruijin Hospital Affiliated to Shanghai Jiao Tong University School of Medicine Shanghai China; ^5^ The Second Hospital of Dalian Medical University Dalian China; ^6^ The Second Affiliated Hospital of Nanjing Medical University Nanjing China

**Keywords:** BCL2, Double‐hit lymphoma, FL‐SJC cell line, Follicular lymphoma, MYC

## Abstract

About 5 per cent of follicular lymphoma (FL) cases are double‐hit (DH) lymphomas. Double‐hit follicular lymphoma (DHFL) cell lines can improve our understanding and drug development on FL. But there are only few DHFL cell lines. Here, we established a new *MYC/BCL2* DHFL cell line, FL‐SJC. The cells were obtained from the hydrothorax of a patient with *MYC/BCL2 *DHFL and cultured for 140 passages in vitro. FL‐SJC cells demonstrated CD19^++^, CD20^+^, CD22^++^, HLA‐DR^+^, CD10^+^, CD38^+^, Lambda^+^ CD23^‐^, CD5^‐^ and Kappa^‐^. The chromosome karyotypic analysis confirmed the co‐existence of t(8;22)(q24;q11) and t(14;18)(q32;q21), as well as additional abnormalities involving chromosomes 2 and 3. Fluorescence in situ hybridization analysis (FISH) showed *IGH/BCL2* fusion gene and the *MYC* rearrangement. In addition, the FL‐SJC cells displayed *KMT2D/MLL2* and *CREBBP* gene mutations. After subcutaneous inoculation of FL‐SJC cells, the SCID mice developed solid tumour masses within 6‐8 weeks. FL‐SJC cells were proven to be free of Epstein‐Barr (EB) virus infection and be multidrug‐resistant. In a conclusion, the FL‐SJC cell line has been identified as a novel *MYC/BCL2* double‐hit follicular lymphoma that can be used as a potentially available tool for the clinical and basic research, together with the drug development for *MYC/BCL2* DHFL.

## 1 INTRODUCTION

Follicular lymphoma (FL) which accounts for 20%‐25% of all non‐Hodgkin's lymphoma (NHL) cases is the second most common form of NHL.^1^, ^2^, ^3^ Cytogenetically, 85%‐90% of patients have a typical translocation t(14;18)(q32;q21) that leads to overexpression of *BCL2* protein.^4^, ^5^ Although FL is considered as indolent, most patients are still incurable and 15% to 28% of cases will turn into an invasive phenotype, usually diffuse large B‐cell lymphoma (DLBCL) within 10 years.^2^

Recently, the concept of double‐hit (DH) lymphoma has attracted considerable attention. DH lymphoma is defined as a chromosomal translocation between the *MYC* gene which locates in 8q24.2 and another recurrent oncogene, such as *BCL2, BCL6* or rarely other genes.^6^, ^7^, ^8^ *MYC* is a transcription factor that regulates the expression of several target genes related to the cell cycle, DNA damage repair, metabolism, protein synthesis and stress response.^9^ *BCL2* usually plays an anti‐apoptotic role.^6^ *MYC/BCL2* DH lymphomas represent approximately 60%‐85% of all cases of DH lymphoma.^7^, ^8^ DH lymphoma (all types) is considered to be ‘high‐grade’ B‐cell lymphoma,^6^, ^10^ has an aggressive clinical course, has poor prognosis, and often involves the bone marrow and the central nervous system with a median overall survival no more than 1‐2 years.^10^, ^11^

The studies on the pathogenesis of DH lymphoma depend on well‐validated DH lymphoma cell lines.^12^ The major advantages of cell lines include the probability of unlimited supply, the global availability, the certainty of background and the infinite viable storability in liquid nitrogen. Until 2016, nearly 30 cell lines meet the diagnosis of DH lymphoma, bearing both *MYC* and *BCL2* rearrangement.^13^, ^14^, ^15^, ^16^, ^17^ Among them, most of them were derived from patients with DLBCL, or Burkitt lymphoma (or B‐ALL), while only 4 cell lines were from patients with FL,^13^ including FLK‐1,^3^ FL‐18,^18^, ^19^ SC‐1^20^, ^21^ and TAT‐1.^22^ FLK‐1 holding t(2;8)(p12;q24) and t(14;18)(q32;q21), established in 2001, was found to depend on a follicular dendritic cells. When follicular dendritic cells were removed, FLK‐1 cells stopped growing and eventually died.^3^ So FLK‐1 is unstable and inconvenient as a cell line.^3^ FL‐18 was established in 1985, in which the translocation [t(8;22)(q24;q13) and t(14;18)(q32;q21)] was not verified by fluorescence in situ hybridization (FISH), Southern blot, polymerase chain reaction (PCR) or other method,^13^, ^18^, ^19^ probably due to the previous technical restriction. Only SC1 with t(8;14;18)(q24;q32;q21) ^20^, ^21^ and TAT‐1^22^ with t(8;14;18)(q24;q32;q21) had fully documented genetic background.

Here, we established and characterized a novel lymphoma cell line, FL‐SJC, that held chromosomal abnormalities of t(8;22)(q24;q11), t(14;18)(q32;q21), del2q and del3, as well as gene mutations of *KMT2D/MLL2* and *CREBBP*. This cell line could provide a useful tool for studies on the malignant nature of FL.

## 2 MATERIALS AND METHODS

### 2.1 Patient

**Figure 1:**
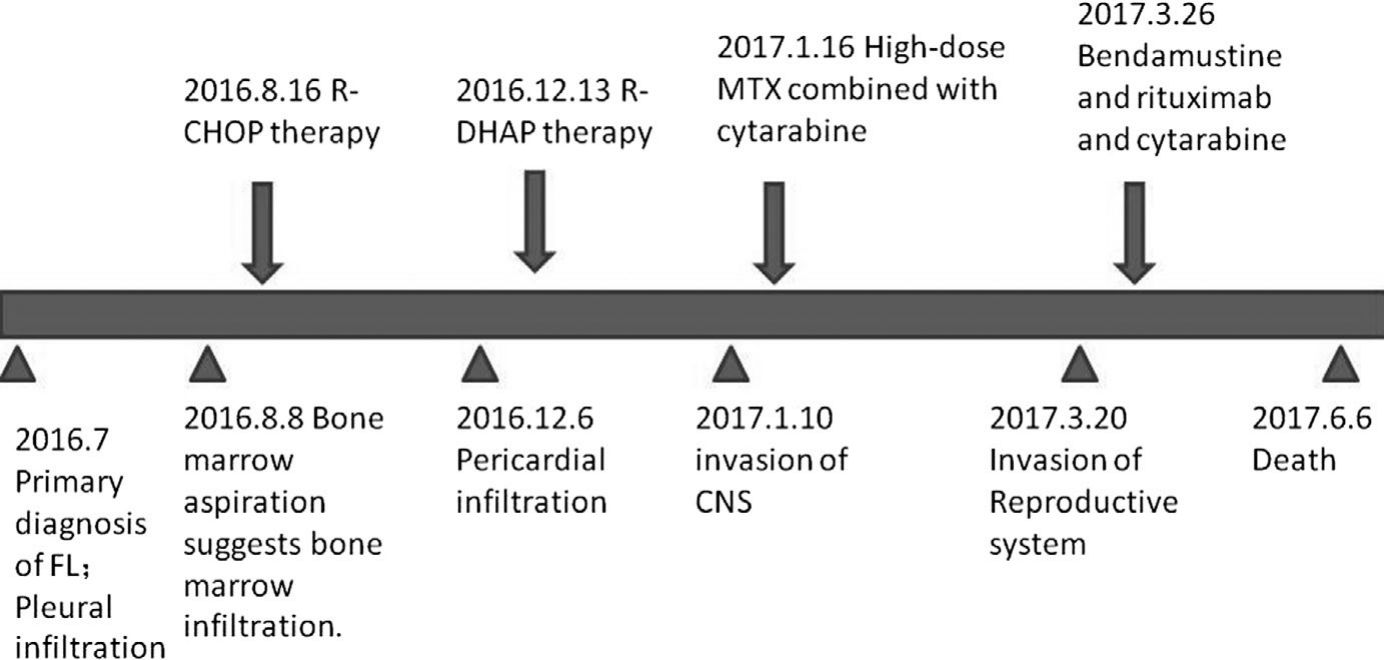
The disease progress and treatment

**Figure 2:**
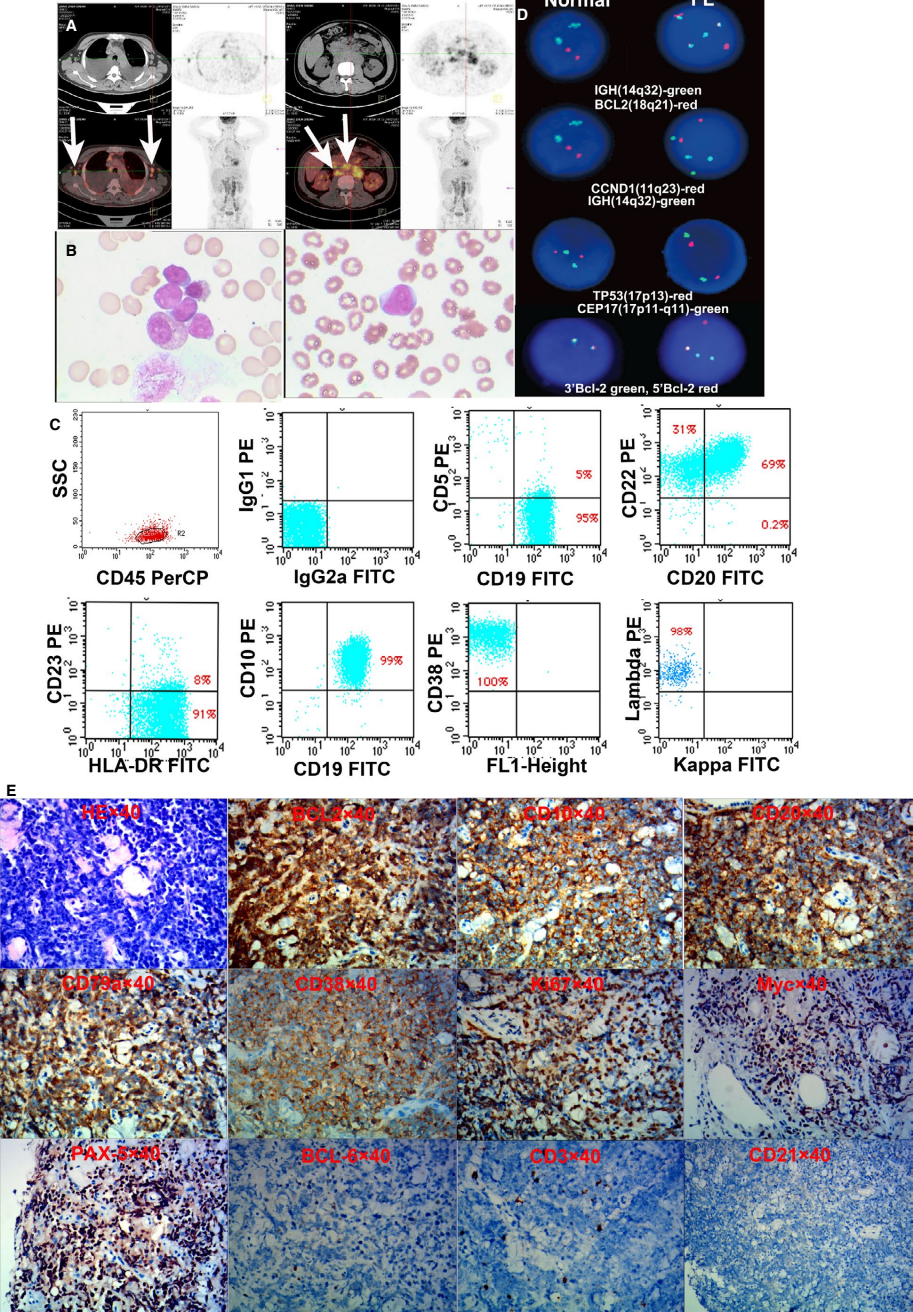
The imaging and laboratory features of lymphoma from the patient. A, PET/CT shows enlarged axillary and retroperitoneal lymph nodes with high SUV_max_ (arrows). The bilateral pleural effusion is also shown. B, Bone marrow smear (left) and peripheral blood smear (right) from the patient show abnormal lymphoma cells with large nuclei and obvious nucleoli (Wright‐Giemsa staining). C, The immunophenotype of original cells from the patient's hydrothorax indicates expression of CD19, CD20, CD22, HLA‐DR, CD10, CD38, and Lambda, but no expression of CD23, CD5 and Kappa. D, FISH results from bone marrow cells show 65% cells have *IGH*(14q32)/*BCL2*(18q21) fusion gene and 64% cells have *BCL2* (18q21) gene break. The fusion genes of *CCND1* (11q23)/*IGH* (14q32) and deletion of *TP53* (17p13) are not found. The multiple copies of IGH are also detected. E, The immunohistology of infiltrated mass near pubic symphysis indicates AE^‐^/AE3^‐^, Vimentin^+^, CD45^+^, BCL2^+^, CD10^+^, CD20^+^, CD79a^+^, CD38^+^, Ki67^+^(75%), c‐Myc^+^, PAX‐5^+^, BCL6^‐^, CD3^‐^, CD21^‐^, CD5^‐^, CyclinD1^‐^, MUM‐1^‐^, CD138^‐^, Kappa^‐^ and Lambda^‐^, suggesting a transformation of DLBCL

A 52‐year‐old male patient with cough, chest tightness, asthma, night sweat, weight loss and enlarged lymph nodes was admitted to our hospital in July 2016 (no fever or chill) (Figure 1). B‐ultrasonic examination showed enlargement of bilateral cervical, supraclavicular, axillary, inguinal and retroperitoneal lymph nodes. Positron emission tomography/computed tomography (PET/CT) showed multiple extensively enlarged lymph nodes with increased maximum of standard uptake value (SUV_max_) as well as spleen and bone infiltration (Figure 2A). Bilateral pleural effusion was also detected (Figure 2A). A right cervical lymph node with high SUV_max_ in PET/CT was excised for biopsy. The biopsy showed BCL2^+^, CD10^+^, CD38^+^(few), CD20^+^, CD43^‐^, Ki67^+^(20%), CD20^+^, BCL6^+^, CD3^‐^, CD5^‐^, CD21^+^, CyclinD1^‐^, SOX‐11^‐^ and ZAP‐70 (few), which fulfilled the diagnostic criteria of follicular lymphoma with grade II. Bone marrow smear revealed 21% of lymphoma cells, and peripheral blood smear revealed 2% of lymphoma cells (Figure 2B). These cells had large nuclei with obvious nucleoli (Figure 2B). Thoracentesis was performed, and pleural effusion was collected. The cells from pleural effusion expressed typical immunophenotype for follicular lymphoma (Figure 2C, see below). Conventional chromosome karyotype analysis of bone marrow cells confirmed the existence of t(14;18)(q32;q21) and t(8;22)(q24;q11) (see below). FISH of mononuclear cells from patient's pleural effusion confirmed *IGH*(14q32)/*BCL2*(18q21) fusion gene (Figure 2D, see below). The laboratory examination showed β_2_‐microglobulin (β_2_‐MG) 5.12mg/L (0.9 ~ 2.7 mg/L), lactate dehydrogenase (LDH) 188U/L(135 ~ 266 U/L), ferritin >2000 μg/L (21.81 ~ 274.6 μg/L), haemoglobin 95 g/L(130 ~ 170 g/L), platelet count 108 × 10^9^/L (100 ~ 300×10^9^/L) and EB virus DNA 1.94 × 10^4^copies/mL (<5 × 10^2^ copies/mL). According to the biopsy, clinical symptom and PET/CT, the patient was diagnosed as follicular lymphoma with grade II, stage IV and B group. According to Follicular Lymphoma International Prognostic Index‐2 (FLIPI‐2), the patient was in high risk.

From August 2016, the patient was treated with R‐CHOP therapy (rituximab, cyclophosphamide, epirubicin, vincristine and prednisone) for four cycles. But the disease did not achieve remission with progressive pleural effusion and emerging pericardial infiltration. The second‐line chemotherapy regimen of R‐DHAP (rituximab, cisplatin, high‐dose cytarabine and dexamethasone) was performed in December 2016. Subsequently, the patient complained with headache and a large number of lymphoma cells were found in the cerebrospinal fluid. An invasion of central nervous system was confirmed. High‐dose methotrexate and cytarabine combined with bendamustine were given since January 2017. However, he suffered from an epileptic seizure frequently and the lymphoma gradually invaded into the reproductive system. A puncture on the mass located between testis and pubic symphysis for biopsy was performed in June 2017. The biopsy showed FL transforming into germinal centre B cell–like (GCB) DLBCL with AE^‐^/AE3^‐^, Vimentin^+^, CD45^+^, BCL2^+^, CD10^+^, CD20^+^, CD79a^+^, CD38^+^, Ki67^+^(75%), c‐Myc^+^, PAX‐5^+^, BCL6^‐^, CD3^‐^, CD21^‐^, CD5^‐^, CyclinD1^‐^, MUM‐1^‐^, CD138^‐^, Kappa^‐^ and Lambda^‐^(Figure 2E). The laboratory examination showed β_2_‐MG 5.4 mg/L, LDH 1262 U/L, white blood cell 2.1 × 10^9^/L, haemoglobin 62 g/L and platelet count 37 × 10^9^/L. Although intensive chemotherapy regimens were chosen, the patient died in June 2017 (Figure 1).

### 2.2 Cell culture

The pleural effusion was collected through thoracentesis in August 2016. The cells were harvested and cultured in RPMI 1640 medium with 10% foetal bovine serum and 1% penicillin/streptomycin without external growth factors or stimulatory cytokines. The cells passaged for at least 140 times were named as the ‘FL‐SJC’.

### 2.3 Cell growth and viability assay

Cell viability of FL‐SJC cell line was assessed using cell counting kit‐8 (Beyotime Biotechnology) according to the directions of the kit. Another two FL cell lines, Dohh2 and SC1 (as a gift generously provided by Prof. Chuanxin Huang, Shanghai Jiao Tong University Collage of Basic Medical Science), were served as controls. The doubling time of these cells was calculated.

### 2.4 Flow cytometry

The original FL cells from pleural effusion and FL‐SJC cells were centrifuged, respectively, and tested for immunophenotypic analysis by flow cytometry. The panel of monoclonal antibodies, including those specific for CD45, CD19, CD20, HLA‐DR, CD19, CD5, CD22, CD23, CD10, CD38, and Kappa and Lambda light chains, was bought from BD Biosciences Co.

### 2.5 Cytogenetic analysis

Chromosome analysis was performed using conventional R‐banding. FISH was performed using a dual‐colour break apart MYC probe, and a dual‐colour break apart IGH probe, as well as IGH(14q32)/BCL2(18q21), CCND1(11q23)/IGH(14q32), and TP53(17p13)/CEP17(17p11‐q11) dual‐colour dual‐fusion translocation probes (Institute of Hematology and Blood Diseases Hospital, Chinese Academy of Medical Sciences and Peking Union Medical College). The cut‐offs to define positive results for rearrangement of *MYC*, rearrangement of *IGH*, rearrangement of *BCL2*, *IGH/BCL2*, *CCND1/ IGH* and *TP53/ CEP17* probes are 2.53%, 4.18%, 2.4%, 2.67%, 2.85% and 2.89%, respectively.

### 2.6 Gene mutation of FL‐SJC cells

Exon sequencing of lymphoma‐associated 38 genes was performed (Institute of Hematology and Blood Diseases Hospital, Chinese Academy of Medical Sciences and Peking Union Medical College). According to the literature,^5^, ^23^ these genes included *ARID1A, ATM, BCL2, BCL6, BIRC3, CCND1, CCND3, MYC, CREBBP, DNMT3A, ECT2L, EP300, EPHA7, EZH2, FBXW7, IDH2, IKZF1, JAK1, JAK2, JAK3, KIT, MLL2, MUM1, MYD88, NOTCH1, NOTCH2, PAX5, PDGFRB, PHF6, PTEN, RELN, TEL, TET2, TNFAIP3, TP53, WHSC1, WT1* and *XPO1*.

### 2.7 Quantitative RT‐PCR

Total RNA was extracted from FL‐SJC cells, Dohh2, SC1 and K562, using the DNA/RNA/protein co‐extraction kit (TianGen Biotech). Peripheral blood mononuclear cells from healthy volunteers were served as a control. Reverse transcription was performed using PrimeScript™ RT Master Mix (TaKaRa RR036A, Otsu, Japan) according to the manufacturer's instructions. The qPCR was performed using the SYBR Premix Ex Taq (TaKaRa RR420A) according to the manufacturer's protocol. GAPDH was used as an internal reference for normalization, and the relative expression of MLL2 mRNA was evaluated by the 2^−ΔΔ^ *^C^* ^T^ method. The following primers according to the literature^24^, ^25^ synthesized by Sangon Biotech (Shanghai, China) were used: GAPDH (forward) 5′‐GGTGAAGGTCGGAGTCAACG −3′and (reverse) 5′‐CTCGCTCCTGGAAGATGGTG −3′; MLL2 (forward)5'‐CGCGGATCCATGCTGCGCCGCGCTCTGCT‐3' and (reverse) 5'‐CCGGAATTCTTACAGTTCATCTTTCACAGCTTTCTG‐3'.

### 2.8 Western blot

The FL‐SJC cells were lysed using cell lysates. Peripheral blood mononuclear cells from healthy volunteers were served as a control. The boiled proteins were electrophoresed with 10% SDS‐PAGE and then transferred onto PVDF membrane. Proteins were detected using the rabbit anti‐MLL2 polyclonal antibody (sc‐292359) purchased from Santa Cruz Biotechnology, Inc, and mouse anti‐β‐actin monoclonal antibody (mAbcam8226) purchased from Abcam, followed by HRP‐conjugated secondary antibodies. Immunoreactive bands were detected by ECL. The signal intensity of the respective bands was measured by the NIH Image J software.

### 2.9 Xenograft transplantation in vivo

FL‐SJC cells (10 × 10^6^) were inoculated into the left armpits of 4‐week‐old female SCID mice (Gempharmatech Co., Ltd) by subcutaneous injection (n = 3). Animals were marked and checked every two weeks for lump formation. After 6 to 8 weeks, solid tumour mass under the armpits from all of the 3 mice inoculated with FL‐SJC cells was detected. The mice were sacrificed under euthanasia, and tumour masses were resected for biopsy. All animal experiments have been reviewed and approved by the Institutional Animal Care and Use Committee of Jiangsu University.

### 2.10 Epstein‐Barr (EB) virus detection

Genomic DNAs from FL‐SJC cells were extracted to detect whether the cells were infected by the EBV or not by PCR. Raji cells (received as a gift from Nanjing Medical University, Nanjing, China) were used as a positive control and Jeko‐1 cells (as a gift from Prof. Xiaodong Xi of Ruijin Hospital, Shanghai Jiao Tong University School of Medicine) were used as a negative control. The primers for the detection of EBV were as follows: EBNA1‐F: GGTAGAAGGCCATTTTTCCAC; EBNA1‐R: CTCCATCGTCAA AGCTGCAC; EBNA2‐F: CAGGTACATGCCAACAACCTT; EBNA2‐R: CCAACAAAG ATTGTTAGTGGAAT.^14^ For the internal control, a PCR of β‐actin was performed. The primers for the detection of β‐actin were as follows: β‐actin‐F: CTCCATCCTGGCCTCGCT GT; β‐actin‐R: GCTGTCACCTTCACCGTTCC.

### 2.11 Drug‐resistance assay

The cell counting kit‐8 was utilized to assess chemosensitivity of FL‐SJC cell lines. The anticancer drugs, including cytarabine (Ara‐c), methotrexate (MTX) and cyclophosphamide (CTX), were added at various concentrations. The absorbance, defined as optical density (OD), was detected with a microplate reader at a wavelength of 450 nm. Inhibition rate was defined as. Dohh2 and SC1 cells were served as controls.

### 2.12 Statistical analysis

Statistical analyses were performed using the GraphPad Prism 5.0. The measurement data were expressed as mean ± standard error. The t test and the analysis of variance were used as appropriate. *P* < .05 indicates that the difference is statistically significant.

## 3 RESULTS

### 3.1 Establishment of the FL‐SJC cell line

Original cells were obtained from the patient's hydrothorax and cultured without any external stimuli. The cells proliferated stably with a doubling time of 24 hours. The cells demonstrated non‐adherent growth without cellular clump formation.

**Figure 3:**
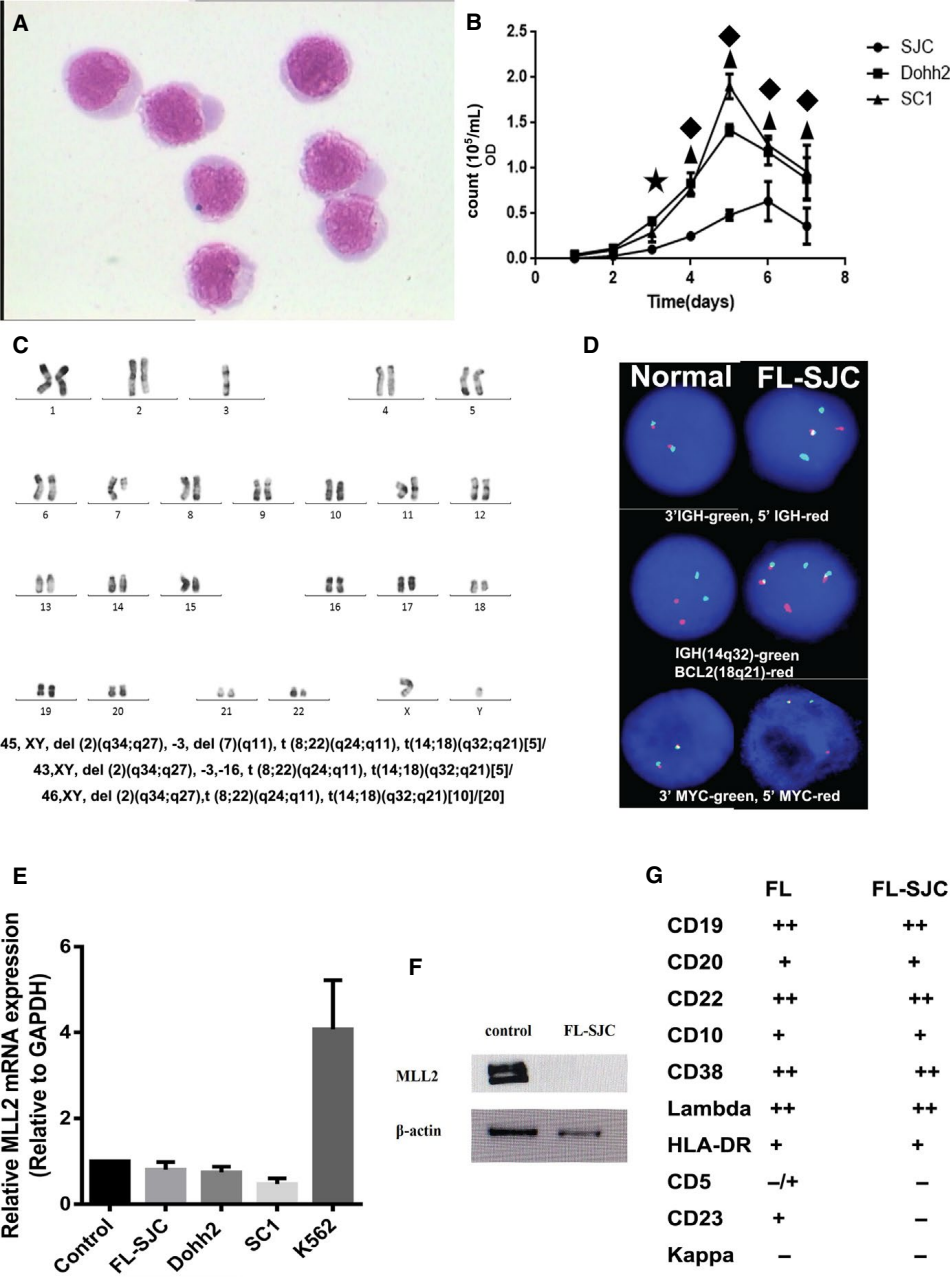
The characteristics of FL‐SJC of 140th generation. A, Smear of FL‐SJC shows several lymphoblast cells with high ratio of nuclear to cytoplasm (Wright‐Giemsa staining). B, The growth and viability of FL‐SJC cells. ▲*P* < .01 for FL‐SJC vs Dohh2. ★*P* < .05 for FL‐SJC vs Dohh2. ◆ *P* < .01 for FL‐SJC vs SC1. C, The chromosome karyocyte analysis confirms the t (8;22)(q24;q11) and t(14;18)(q32;q21) suggesting that the FL‐SJC is a new double‐hit lymphoma cell line. D, FISH analysis confirms IGH break and translocation between *IGH* and *BCL2*, and *MYC* break in the FL‐SJC cells. E, MLL2 mRNA level of FL‐SJC cells is quantified using RT‐PCR. The mononuclear cells in peripheral blood from healthy volunteers are served as controls. The expression of MLL2 in FL‐SJC is similar to that in the normal control cells (*P* > .05). Dohh2, SC1 and K562 cells are also tested. F, Western blot result shows that the MLL2 protein expression in FL‐SJC cells is decreased compared with that in the normal control cells. G, The immunophenotype of original FL cells and FL‐SJC cells

FL‐SJC cells were small‐to‐medium sized and blast‐like lymphocytes, and the cytoplasm was moderately rich and basophilic (Figure 3A). The large nuclei were round to ovoid with rough chromatin and obvious nucleoli (Figure 3A). The morphologic features of FL‐SJC cells were similar to that of original FL cells (Figure 2B).

The viability of FL‐SJC cells was high, while a proliferative rate was not as high as that of Dohh2 and SC1 cells (Figure 3B).

### 3.2 Immunophenotypic characterization

Original FL cells from the patient's hydrothorax expressed CD19, CD20, CD22, CD10, CD38, Lambda and HLA‐DR, partially expressed CD5 and CD23, but did not express Kappa (Figure 2C, Figure 3G). FL‐SJC cells were positive for CD19, CD20, CD22, CD10, CD38, Lambda and HLA‐DR, and were negative for CD5, CD23 and Kappa (Figure 3G).

### 3.3 Chromosome karyotyping and FISH analysis

Conventional chromosome karyotype analysis of FL cells from patient's bone marrow revealed 44X, ‐Y, −22, t(8;22)(q24;q11), t(14;18)(q32;q21) [3]/46XY [7], while FL‐SJC cells demonstrated a complex karyotype: 45,XY, del(2)(q34;q27),‐3,del(7)(q11),t(8;22)(q24;q11),t(14;18)(q32;q21)[5]/ 45,XY, del(2)(q34;q27),‐3, −16,t(8;22)(q24;q11),t(14;18)(q32;q21)[5]/ 45,XY, del(2)(q34;q27), t(8;22)(q24;q11),t(14;18)(q32;q21)[10]/[20] (Figure 3C). The karyotype of FL‐SJC cells was similar to but more complex than the original FL cells.

FISH of original FL cells from patient's pleural effusion showed 65% cells had *IGH*(14q32)/*BCL2*(18q21) and 64% cells had *BCL2* (18q21) gene rearrangement. The fusion genes of *CCND1* (11q23)/*IGH* (14q32) and deletion of *TP53* (17p13) were not found (Figure 2D). The multiple copies of *IGH* were also detected (Figure 2D). FISH of FL‐SJC cells showed that 95% cells had *IGH* (14q32)/*BCL2*(18q21) (the cut‐off value was 2.67%) fusion gene, and 96% cells had *BCL2*(18q21) gene rearrangement (the cut‐off value was 4.18%). 96% cells had *IGH*(14q32) gene rearrangement, 90% cells had a *MYC*(8q24) gene rearrangement most likely involving the *IGL* gene in 22q11 region according to the result of conventional cytogenetic analysis. The proliferation of *IGH* was also detected (Figure 3D). The detection rate of FISH was higher in FL‐SJC cells than that in FL cells, probably due to the purification of long culture.

### 3.4 Gene mutation of FL‐SJC cells

FL‐SJC cells had *MLL2* (Exon14, c.4135_4136del, p.M1379Vfs*52) and *CREBBP* (Exon30, c.5039_5041del, p.S1680delS) gene mutations. The other gene mutations were also detected, including *CREBBP* (Exon25, c.4174C > T, p.R1392X), *CCND3* (Exon5, c.869_872dup, p.L292Tfs*56), *BCL2* (Exon2, c.256C > T, p.L86F), *RELN* (Exon43, c.6575G > A, p.R2192Q), *MYC* (Exon2, c.221C > T, p.P74L) and *NOTCH1* (Exon11, c.1837C > T, p.R613C) (Table 1).

**Table 1:** Gene mutation of FL‐SJC cells

Gene	Transcript	Site	DNA	Amino acid	Mutation ratio (%)
*KMT2D/MLL2*	NM_003482	Exon14	c.4135_4136del	p.M1379Vfs*52	45.8
*CREBBP*	NM_004380	Exon30	c.5039_5041del	p.S1680delS	47.5
*CREBBP*	NM_004380	Exon25	c.4174C > T	p.R1392X	51.1
*CCND3*	NM_001760	Exon5	c.869_872dup	p.L292Tfs*56	38
*BCL2*	NM_000633	Exon2	c.256C > T	p.L86F	39.9
*RELN*	NM_005045	Exon43	c.6575G > A	p.R2192Q	48.5
*MYC/MYC*	NM_002467	Exon2	c.221C > T	p.P74L	61.2
*NOTCH1*	NM_017617	Exon11	c.1837C > T	p.R613C	49.7

### 3.5 mRNA level and protein expression of MLL2

The quantitative RT‐PCR results showed that mRNA level of *MLL2* was significantly up‐regulated in K562 compared with that in the normal control cells (*P* < .05), consistent with the previous report.^26^ However, mRNA expression of *MLL2* in FL‐SJC, as well as other two follicular lymphoma cell lines (Dohh2 and SC1), had no statistically significant difference (*P* > .05) compared with the normal control (Figure 3E). The protein expression of *MLL2* in FL‐SJC cells was decreased compared with that in normal control cells (Figure 3F).

### 3.6 Xenotransplantation of FL‐SJC cells into SCID mice

**Figure 4:**
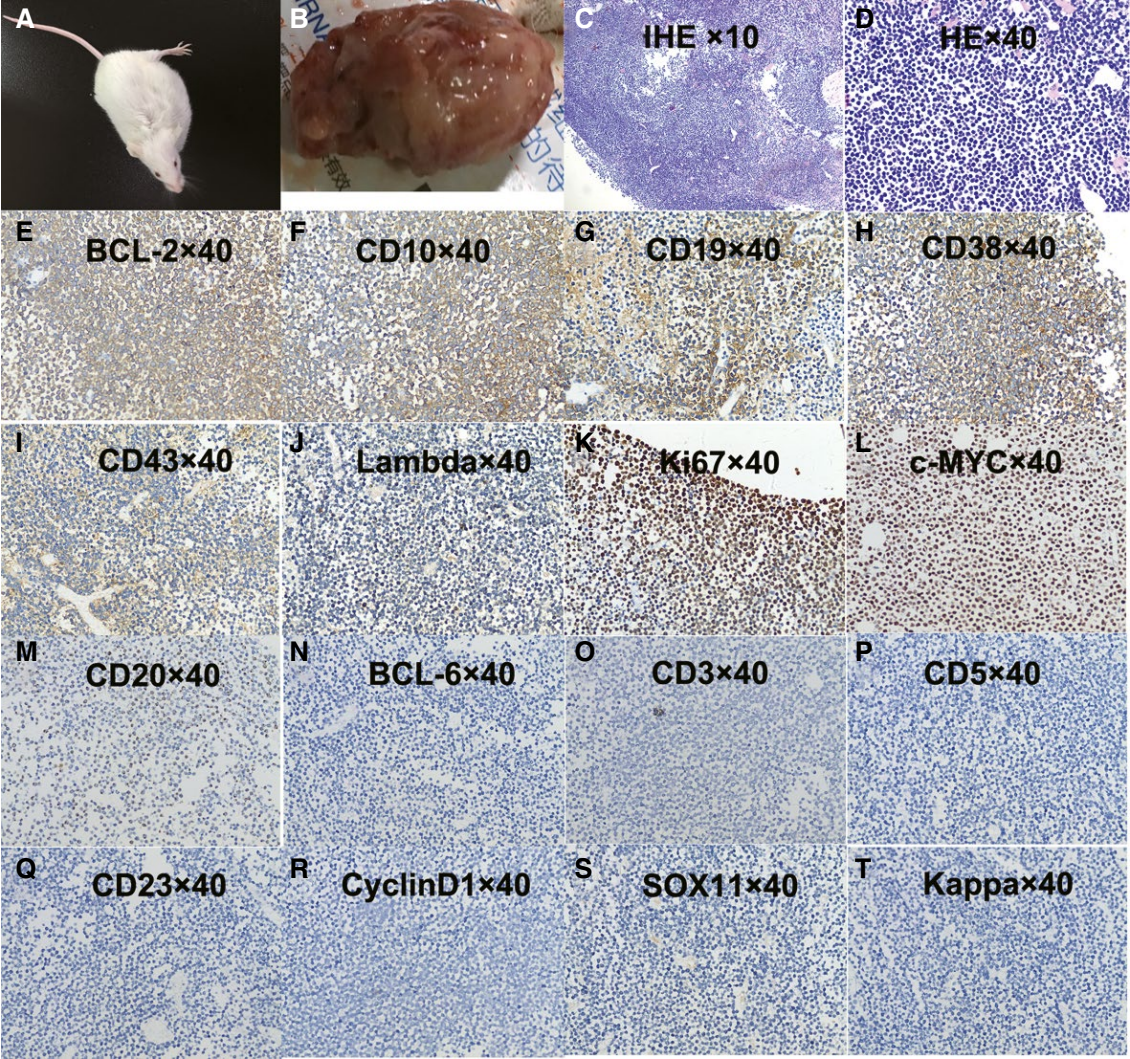
Transplantation of FL‐SJC cells into SCID mice. A, The SCID mouse that received a transplant of FL‐SJC cells. B, The tumour mass from the mouse. C and D, H&E paraffin sections of the tumour mass. E‐T, Immunohistochemical analysis of the transplanted lymphoma from a SCID mouse showing BCL2^++^, CD10^++^, CD19^++^, CD38^++^, CD43^+^, Lambda^+^, Ki67^++^, MYC^++^, CD20^±^, BCL6^‐^, CD3^‐^, CD5^‐^, CD23^‐^, CyclinD1^‐^, SOX‐11^‐^ and Kappa^‐^

FL‐SJC cells (10 × 10^6^) were seeded into the left armpits of SCID mice by subcutaneous injection. Within 6‐8 weeks, all the mice (3 mice) gradually developed into solid tumour masses in the armpits (Figure 4A). Further biopsy analysis showed that the tumour mass removed (Figure 4B) consisted of lymphocytes from human B cell, morphologically consistent with FL‐SJC cells. Immunohistology results showed BCL2^++^, CD10^++^, CD19^++^, CD38^++^, CD43^+^, Lambda^+^, Ki67^++^, MYC^++^, CD20^±^, BCL6^‐^, CD3^‐^, CD5^‐^, CD23^‐^, Cyclin D1^‐^, SOX‐11^‐^ and Kappa^‐^ (Figure 4C‐T).

### 3.7 EBV status in FL‐SJC cells

**Figure 5:**
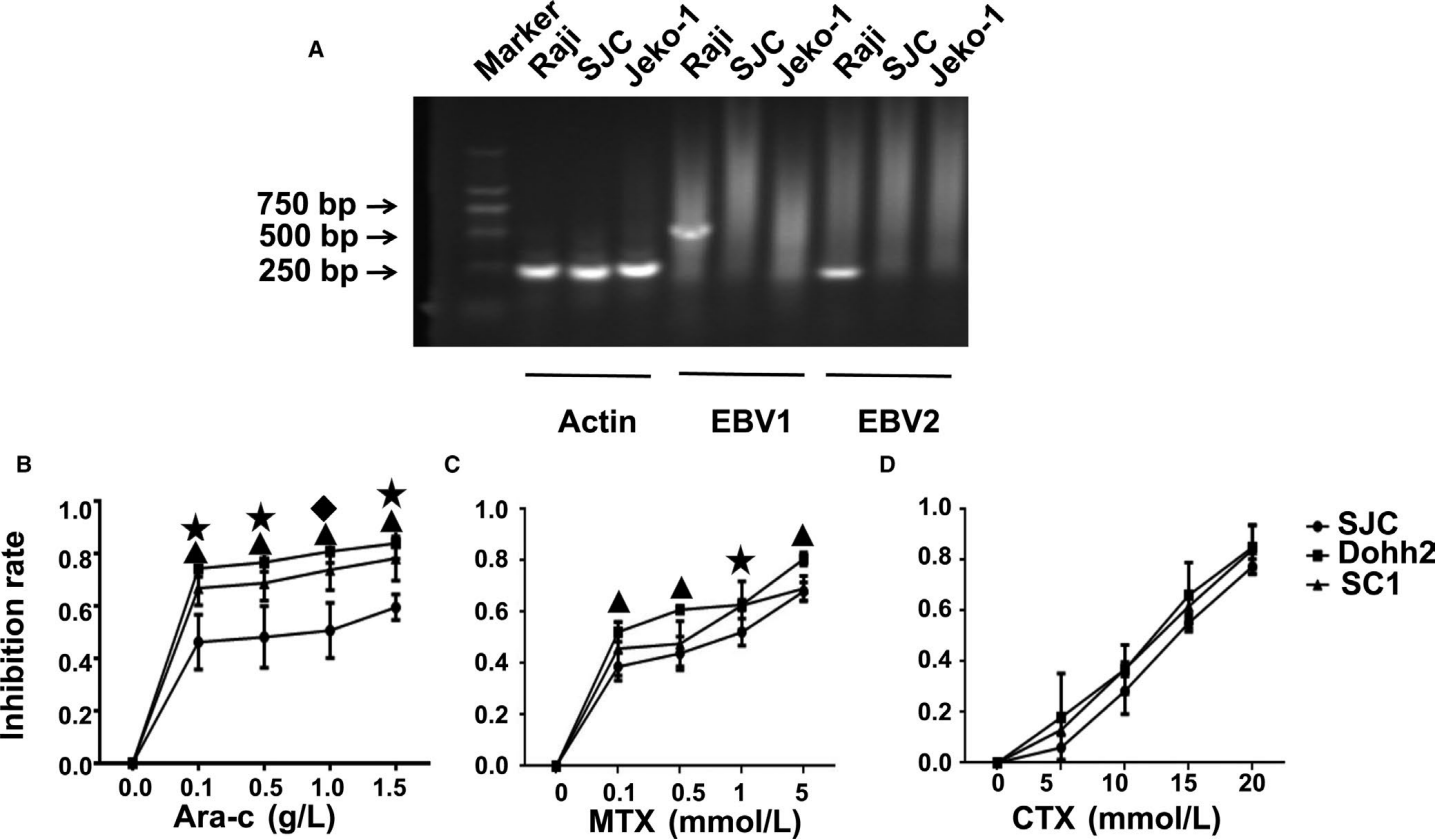
EB virus test and drug resistance of FL‐SJC cells. A, Test of EB virus. PCR result shows that the FL‐SJC cells are not infected with EB virus. B‐D, Comparative drug‐resistance profiles in the FL‐SJC, Dohh2 and SC1. B, Inhibition rate of Ara‐c in 3 cell lines. ▲*P* < .01 for FL‐SJC vs Dohh2. ★*P* < .05 for FL‐SJC vs SC1. ◆ *P* < .01 for FL‐SJC vs SC1. C, Inhibition rate of MTX in 3 cell lines. ▲*P* < .01 for FL‐SJC vs Dohh2. ★*P* < .05 for FL‐SJC vs Dohh2. D, Inhibition rate of CTX in 3 cell lines. *P* > .05 for FL‐SJC vs Dohh2 and SC1

PCR analysis showed EBV negative in FL‐SJC cells. As expected, the genomes of EBV1 (type 1) and EBV2 (type 2) were detected in the Raji cell line (as positive control), but not in the Jeko‐1 cell line (as negative control) (Figure 5A).

### 3.8 Drug resistance

The chemosensitivity of FL‐SJC, Dohh2 and SC1 to Ara‐c, MTX and CTX is shown in Figure 5B, 5C and 5D. The inhibition rate of Ara‐c in FL‐SJC was lower than that in Dohh2 and SC1 and the inhibition rate of MTX in FL‐SJC was lower than that in Dohh2, suggesting that FL‐SJC demonstrated a high drug resistance.

## 4 DISCUSSION

Although therapeutic outcomes of FL have markedly improved in the last few decades, 15% to 28% of cases will transform into an aggressive phenotype, typically DLBCL.^1^, ^2^ The therapeutic approaches for FL are most often based on stage, FLIPI and burden of disease.^1^, ^6^ In the beginning, the disease was diagnosed as FL with grade II, stage IV and B group, and with high risk FLIPI. The patient showed an unsatisfactory outcome associated with normal chemotherapy including R‐CHOP, R‐DHAX and high‐dose MTX. Autologous hematopoietic stem cell transplantation should have been performed; unfortunately, the patient died during the induction treatment.

The aggressive characteristics of disease may result from the gene rearrangements. In this patient, conventional chromosome karyotype analysis and FISH examination confirmed the co‐existent of t(14;18)(q32;q21) and t(8;22)(q24;q11) involving gene rearrangement of *MYC/BCL2*, which recently is named as ‘double hit (DH)’. The ‘DH’ lymphomas are defined as a chromosomal breakpoint affecting the *MYC*/8q24 and another recurrent genetic abnormality, mainly involving *BCL2, BCL6, BCL3* or other genes.^27^ Among them, most are *MYC/BCL2. MYC/BCL2* double‐hit lymphomas tend to demonstrate themselves with advanced stage and poor prognostic parameters including extranodal infiltration and elevated LDH,^10^, ^27^ and a high FLIPI score. This patient had high LDH, infiltration in the bone marrow, reproductive system and the central nervous system. All these features were fulfilled in this patient. Most common transformation of DHFL is transformation into DLBCL^2^; the biopsy from infiltrated mass near the pubic symphysis in this patient confirmed the DLBCL transformation at the late stage of disease.

One of main limitations of the successful treatment of *MYC/BCL2* DH lymphoma is lack of comprehensive understanding of disease pathogenesis and mechanisms of chemotherapeutic resistance, as well as knowledge of potential therapeutic targets. Cell lines are critical for mechanism exploration and drug discovery.^28^, ^29^

However, as far as we know, there have been only a few published manuscripts describing the establishment and characterization of defined DHFL cell lines.^3^, ^13^, ^18^, ^19^, ^20^, ^21^, ^22^ Some of them are not in full detail in genetic messages.^13^, ^18^, ^19^ In this report, we established a novel ‘double‐hit’ follicular lymphoma cell line, FL‐SJC, which we wish as a useful tool for the improved understanding of *MYC/BCL2* ‘double‐hit’ FL.

The success rate for the establishment is low, and it is impossible to predict whether it is successful for specimens from source.^12^ Those lymphoma cell lines which were established successfully were high‐grade lymphomas ^30^ and from the ascitic fluid, or pleural fluid.^4^, ^20^, ^21^ FL‐SJC cell line was from the pleural effusion and passaged for at least 140 times. It took us 2 years to establish it.

We then identified the cell line according to the published guidelines.^31^, ^32^ The clinical characteristics and cell culture data, as well as immunological and cytogenetic features were described. Established FL‐SJC cells demonstrated small‐medium size, blast‐like morphology.

The biopsy from initial lymph node fulfilled the immunophenotype of follicular lymphoma.^33^ The biopsy from late infiltrated mass near the pubic symphysis confirmed FL transforming into DLBCL. The positive rate of Ki67 gradually increased suggesting aggressive conversion. The FL‐SJC cells expressed CD19, CD10, CD22, HLA‐DR, CD38, Lambda and CD20, but did not express CD23, CD5 and Kappa, which was similar to that of the original FL cell from pleural effusion. The FL‐SJC cells were fully negative for CD23 and CD5 which were 8% and 5% positive, respectively, in primary FL cell, suggesting that the FL‐SJC cells have a higher purity than original tumour cells.

Xenotransplantation of FL‐SJC cells into SCID mice can cause the solid tumour mass, suggesting the high proliferative viability. The immunohistology of tumour showed the loss of CD20. Pham LV et al ever reported that the RC cell line showed gradually reduced CD20 expression with the culture.^14^ Denyssevych T et al also observed that the expression of the CD20 antigen on the surface of Tat‐1 cells grown in mice gradually decreased [23]. It is possibly due to selection of more immature lymphoma cells for its growth advantage during the culture and inoculation, because CD20 is mainly expressed on mature B lymphocytes.

FISH and karyotypic analysis confirmed the existence of t(8;22)(q24;q11) and t(14;18)(q32;q21) double fusion genes. Chromosomal translocations join *MYC* in 8q24 to the light chain *IG* gene 22q11(Lambda light chain) and *BCL2* in 18q21 to the heavy chain *IG* gene 14q32.^27^ Unfortunately, the test of *IGL/MYC* gene fusion was not confirmed due to unavailability of dual‐fusion translocation probes. The FL‐SJC hold more chromosome change than FL did, having deletion of chromosome 2(q34;q27) and 3.

Additionally, recently some genes had been reported to be associated with the prognosis of lymphoma.^23^ We tested the prognosis‐related genes. *EZH2* mutation which was associated with good prognosis, according to the newest risk prognostication developed in FL, the M7‐FLIPI,^23^ was not found in FL‐SJC, while *CREBBP* (a histone modification enzyme) mutation, which was associated with poor prognosis,^23^ was found in FL‐SJC *MLL2*, also known as *KMT2D* or *ALR*, is another histone modification enzyme, gene mutations of which are mainly found in lymphomas (especially DLBCL and FL from indolent to aggressive) and myeloma.^34^ *MLL2* acts as a central tumour suppressor in FL.^35^ The frameshift mutation of *MLL2* gene, which has 54 exons, also was found in Exon14 in FL‐SJC cells. By RT‐PCR, we found mRNA level of *MLL2* in FL‐SJC cells was similar to that in normal control cells. While MLL2 protein expression was obviously decreased in FL‐SJC cells by Western blot. The anti‐MLL2 antibody (sc‐292359) we used is raised against amino acid 4021‐4320 mapping which is at the downstream of mutation site. It is possible that the frameshift mutation of *MLL2* in FL‐SJC cells causes a stop of translation from mRNA (20635bp) to protein. In this study, we only tested 38 genes, in the future we will perform RNA‐sequencing to show the global changes of gene expression and find some useful clues.

The expression of Epstein‐Barr virus associated antigen (EBNA‐I) is often detected in established lymphoma lines.^30^ EBV can immortalize cell lines^36^ and truly malignant cell lines must be distinguished from EBV‐immortalized normal cells.^12^ This FL‐SJC cell line was negative for EBV according to PCR result suggesting that the immortality of FL‐SJC results from intrinsic gene changes.

We had made a preliminary estimation of FL‐SJC sensitivity to chemotherapeutic drugs, Ara‐c, MTX and CTX. FL‐SJC demonstrated a higher resistance to Ara‐c than Dohh2 and SC1 did, and to MTX than Dohh2 did. This may reflect subtle changes in cell growth regulation.

## 5 CONCLUSION

We described the establishment and characterization of a new *MYC/BCL2* DHFL cell line, FL‐SJC, which would be useful in studies on human DHFL, and therapeutic drug development.

## CONFLICT OF INTEREST

The authors declare that they have no conflict of interest.

## AUTHOR CONTRIBUTION

**Min Chen:** Formal analysis (lead); Methodology (lead); Writing‐original draft (equal). **Guoxiong Jiang:** Formal analysis (equal); Methodology (equal); Writing‐original draft (equal). **Yichen Liu:** Formal analysis (equal); Methodology (equal); Writing‐original draft (equal). **Dongya Li:** Methodology (equal). **Tiantian Li:** Methodology (equal). **Jie Peng:** Methodology (equal). **Qian Jiang:** Formal analysis (equal); Methodology (supporting); Software (equal). **Haiyan You:** Formal analysis (equal); Methodology (supporting); Software (equal). **Rong Ba:** Formal analysis (equal); Methodology (supporting); Software (equal). **Jinlan Pan:** Formal analysis (equal); Methodology (supporting). **Mei Li:** Formal analysis (equal); Methodology (supporting). **Weiguo Long:** Formal analysis (equal); Methodology (supporting). **Jinsong Yan:** Writing‐review & editing (equal). **Yan Zhu:** Investigation (equal); Resources (equal). **Yun Wang:** Methodology (supporting); Software (equal). **Xiaodong Xi:** Formal analysis (equal); Methodology (equal); Writing‐review & editing (equal). **Jianhua Mao:** Conceptualization (equal); Data curation (equal); Methodology (equal); Supervision (equal); Writing‐original draft (equal). **Xiaofeng Shi:** Conceptualization (lead); Data curation (equal); Funding acquisition (lead); Investigation (lead); Methodology (equal); Resources (lead); Supervision (lead); Writing‐original draft (lead); Writing‐review & editing (lead).

## Data Availability

The data that support the findings of this study are available from the corresponding author upon reasonable request.
